# Supplementary material for: Reconstitution of SPO11-dependent double-strand break formation
Source: Nature. 2025 Feb 19;639(8055):784–91. doi: 10.1038/s41586-025-08601-2 (PMC11922745; doi:10.1038/s41586-025-08601-2)
Supplement: Supplementary file 1 — This file contains Supplementary Discussion, Supplementary Figs. 1 and 2, Supplementary Tables 1 and 2 and Supplementary References. [file 41586_2025_8601_MOESM1_ESM.pdf]

nature portfolio

https://doi.org/10.1038/s41586-025-08601-2

Supplementary information

Reconstitution of SPO11-dependent double-strand break formation

In the format provided by the authors and unedited

Supplementary Information for:

Reconstitution of SPO11-dependent double-strand break formation

Zhi Zheng, Lyuqin Zheng, Meret Arter, Kaixian Liu, Shintaro Yamada, David Ontoso, Soonjoung Kim, and Scott Keeney

Correspondence to: s-keeney@ski.mskcc.org

This pdf contains:

Supplementary Discussion
Supplementary References
Supplementary Tables 1 and 2
Supplementary Figure 1: Gel source data
Supplementary Figure 2: Gallery of AlphaFold3 models

Other Supplementary Materials for this manuscript include the following:

Supplementary File 1. AlphaFold3 model of SPO11–TOP6BL dimer bound to DNA (.pdb file)
Supplementary File 2. Protein sequences (.pdf file)
Graph Source Data (individual Excel files)

Supplementary Discussion

1. Prior biochemical studies of SPO11, TOP6BL, or SPO11–TOP6BL complexes

Previous attempts to purify recombinant proteins from various species after expression in *Escherichia coli* often resulted in poor solubility[65-68]. Purified plant SPO11 orthologs by themselves have moderate affinity for binding to duplex DNA (apparent K_d of 0.3 to 0.5 μM), but the only reported instance of DNA cleavage activity in vitro — for one of the five SPO11 homologs present in rice — did not involve covalent protein attachment to the DNA and was not shown to require the catalytic tyrosine or other active site residues[66].

Recombinant mouse TOP6BL and its ortholog from *Arabidopsis thaliana* (MTOPVIB) were also purified alone after expression in insect cells[26,69]. Proteins from both species showed modest DNA binding (apparent K_d of 0.6 to >1.5 μM for the mouse protein, depending on the DNA substrate; ~0.15 μM for the *A. thaliana* protein). The *A. thaliana* protein showed no detectable binding to ATP and was reported to be dimeric in solution. By contrast, mouse TOP6BL was monomeric in solution but formed (likely nonphysiological) dimers during purification via disulfide crosslinking.

Mouse SPO11–TOP6BL complexes were purified after expression in *E. coli*, but it was not clear if the material retained biologically relevant activity such as DNA binding or DNA cleavage[9]. The purified mouse proteins were suggested to form 2:2 complexes on the basis of low-resolution SEC and glycerol gradient sedimentation analyses, but misfolding or nonspecific aggregation could not be excluded. Complexes of *A. thaliana* MTOPVIB with SPO11-1 and SPO11-2 have also been purified, but the complexes had DNA-binding properties indistinguishable from MTOPVIB alone and did not cut DNA[69].

Although many aspects of DSB formation are evolutionarily conserved, there are also key differences[70]. For example, there is no evidence for a role for Ski8 homologs outside of fungi[71] and no clear orthologs of Rec104 outside of the family Saccharomycetaceae[24]. Moreover, the amino acid sequence, tertiary structure, and domain composition of the Top6B homologs in yeast and mouse differ strikingly[23,24]. These differences make it important to study SPO11 and its partners in different taxa, particularly in mammals.

2. Evaluating the plausibility of the AlphaFold3 models of dimeric SPO11–TOP6BL complexes bound to DNA

Agreement of models with known aspects of SPO11 complexes. Multiple features of the AlphaFold3 models meet expectations based on empirical structures and functional data for homologous eukaryotic and archaeal proteins. Taken together, these findings suggest that the AlphaFold3 models are plausible representations of dimeric, DNA-bound, pre-DSB complexes.

First, the overall protein architecture closely resembles Topo VI holoenzyme crystal structures[14,15] (**Extended Data Fig. 3f**), with the SPO11–TOP6BL interface in particular matching well with the Top6A–Top6B interface (**Extended Data Fig. 3g**). When individual protein domains are aligned in isolation, the SPO11 WH and Toprim domains align well with crystal structures of archaeal Top6A (Cα rmsd 1.7 Å for WH domains and 2.0 Å for Toprim domains) and cryo-EM structures of yeast Spo11 (Cα rmsd 11.0 Å for WH domains and 7.8 Å for Toprim domains), and the transducer and GHKL domains of TOP6BL align well with archaeal Top6B (Cα rmsd 14 Å for transducer domains and 13 Å for GHKL domains)[14,15,18,24,32]. Moreover, AlphaFold2 models of TOP6BL alone were previously shown to agree well with small-angle x-ray scattering data[26].

Second, the DNA is positioned across a channel formed by the two SPO11 protomers and embraced by the helical transducer domains of the two TOP6BL protomers (**Fig. 3a,b**), as predicted by models of the Topo VI catalytic cycle[14,15,18,33]. Moreover, the modeled DNA binding

surface on SPO11 corresponds to the cognate surface of yeast Spo11 defined by hydroxyl radical footprinting and cryo-EM structures[24,25].

Additionally, details of the protein-DNA interface agree well between the AlphaFold3 model and the yeast cryo-EM structures (which are thought to mimic a post-DSB configuration[24]). For example, nearly all of the direct DNA contacts appear to involve SPO11, and are consistent with being primarily interactions with the sugar-phosphate backbone (**Fig. 3d and Extended Data Fig. 4a,b**). Moreover, the AlphaFold3 model places the DNA close to the homologs of many of the SPO11 amino acid residues that engage DNA in the yeast cryo-EM structures[24] (**Fig. 3d and Extended Data Fig. 3a–c**). The similarities include two principally basic patches making backbone contacts (**Extended Data Fig. 4a**); involvement of K175 (yeast K173) in the only clear base contacts (albeit to different positions relative to the dyad axis in yeast and mouse) (**Extended Data Fig. 4b**); and positioning in the minor groove of a series of amino acid side chains from diverse structural elements (previously described as “fingers” in yeast Spo11) (e.g., **Extended Data Fig. 4b**). Notably, however, TOP6BL does appear to contribute directly to DNA binding, particularly around the regions of biased base composition near –10 and +10 positions (**Fig. 3d**), potentially consistent with relatively low affinity binding of purified TOP6BL alone to duplex oligonucleotide substrates (K_d > 500 nM)[26].

Third, two Mg^{2+} ions are positioned within each Toprim domain coordinated by residues E224, D277 and D279 (**Fig. 3e**). This is consistent with expectation for the two-metal-ion catalytic site architecture proposed for type II topoisomerases[72-74]; consistent with structures of Top6A and yeast Spo11[18,24]; and consistent with the importance of these acidic residues for yeast Spo11 function in vivo[75]. Moreover, the metal-binding pocket of each Toprim domain is close to Y138 of the other SPO11 monomer to assemble the expected hybrid active sites, and the two catalytic tyrosines lie close to opposite strands of the DNA as expected (**Fig. 3d,e**). Other aspects of the predicted SPO11 dimer interface also match well with crystal structures for Topo VI (**Extended Data Fig. 5a,b**).

Fourth, the DNA is consistently predicted to be bent (108° ± 21°, mean ± SD of 25 models with DNAs of varying sequence and length) (**Fig. 3b,c and Extended Data Fig. 3d,e**). DNA bending was predicted for Topo VI[33] and was observed for mouse and yeast proteins in AFM experiments (**Fig. 1g**)[25] and in a cryo-EM structure of monomeric yeast core complexes bound to DNA containing a ssDNA gap[24].

Symmetry and asymmetry of models. Of the 25 AlphaFold3 models analyzed here, only one model had nearly identical distances from each Y138 to its “scissile” phosphate as defined for a 2-bp offset centered on the DNA bend axis, and only another three models had roughly symmetrical distances that were also small (≤3 Å) (**Extended Data Fig. 3h**). The rest of the models had substantial asymmetry or very long Y138–phosphate distances. The model we selected for detailed display is in the middle of the pack for both symmetry and distance.

DNA sequence. All of the AlphaFold3 models have DNA sequences that contain a match to the preferred base composition, but the bend and the protein active sites were often placed somewhere other than the “right” position on the DNA in the models. For example, the presumptive “scissile” phosphates in the representative model shown are shifted one base pair over from the preferred sequence (**Fig. 3d**). Even for the same input sequence, models differed with respect to the protein position on the DNA. One can see the signature of this variation in the model colored by pLDDT score (**Extended Data Fig. 3a**). The DNA has a low score (red color) because the predicted bend position is variable even though the DNA bending path is highly superimposable (**Extended Data Fig. 3d**).

It thus appears that AlphaFold3 is not reliably detecting the fine-scale sequence determinants that shape SPO11 cleavage probability. This could mean that AlphaFold3 is not

yet able to model the effects of DNA sequence on SPO11 binding and DNA bending, or it could mean that the main effects of sequence are on catalysis and not on DNA binding.

Additional interaction of TOP6BL C terminus with SPO11. Many of the models place a C-terminal helix from TOP6BL near the underside of the SPO11 from the other complex (i.e., $TOP6BL^A$ interacting with $SPO11^B$), preceded by a long linker predicted to be disordered (**Extended Data Fig. 3a,b**). Some of the predicted interfacial residues in SPO11 are conserved in several species, including Y380, V384, and N388, but they are missing entirely from the *S. cerevisiae* protein (**Extended Data Fig. 3b**). This TOP6BL C-terminal helix was previously shown to bind to REC114 using some of the same residues predicted to interact with SPO11 (e.g., W562)[59]. We emphasize that this predicted interaction with SPO11 should be viewed cautiously because it has not been experimentally confirmed, but it raises the interesting possibility that defects in this interaction may contribute to the meiotic deficiencies reported for *Top6bl-W562A* mutant mice[59]. Moreover, because this putative SPO11-TOP6BL interface could stabilize a SPO11 dimer, competition between SPO11 and REC114 for binding this TOP6BL segment might provide means to regulate dimerization.

3. Potential mechanism and implications of SPO11 supercoil relaxation activity

We observe a slow supercoil relaxation activity for SPO11 specifically when nicking had occurred, including conditions where only nicking is possible (e.g., with mixtures of SPO11-Y138F and -E224A mutant proteins). We propose that this topoisomerase activity arises from a swiveling reaction for a dimeric complex stalled at the nicked stage, followed by religation (**Extended Data Fig. 5j**). This would be akin to a type IB topoisomerase's swiveling activity rather than a type IA or type II strand passage activity.

Specifically, we envision that the weak SPO11 dimer interface allows the two SPO11–TOP6BL complexes to rotate relative to one another, swiveling around the uncut DNA strand opposite to the nick. Reestablishment of the dimer interface after swiveling would then allow the tyrosyl phosphodiester and 3′-OH of the cleaved strand to interact and reseal the nick. A nonexclusive alternative is that swiveling could instead involve release of the noncovalently bound SPO11 monomer, which would necessitate rebinding to effect religation. This scenario could also lead to irreversible nicking. Whether or not SPO11-generated nicks accumulate in vivo has not been definitively established, but attempts to detect nicks in *S. cerevisiae* have not yielded positive evidence for them, suggesting that they either do not form in appreciable numbers relative to DSBs, or they have a much shorter lifespan than DSBs[28,30].

In cryo-EM structures, amino acid residues in the yeast Spo11 Toprim domain make direct contacts with the 3′-OH when bound to a DNA end or to a gapped DNA analogous to the product of a nicking reaction[24]. These interactions involve the Toprim domain that would have supported strand cleavage by the catalytic tyrosine on the other Spo11 monomer, i.e., they involve the non-covalently bound Spo11 monomer (**Extended Data Fig. 5j**). Assuming similar contacts for the mouse protein, both SPO11 proteins may remain tightly bound to a nicked DNA: both monomers would have their extensive array of backbone contacts (**Fig. 3d**) supplemented by either covalent phosphotyrosyl attachment for one monomer, or Toprim contacts to the newly generated 3′-OH for the other monomer. If both monomers remain bound, we would further speculate that relaxation activity might be disfavored in vivo because the binding of SPO11 to nucleoprotein condensates of REC114, MEI4 and other factors would inhibit rotation of SPO11 monomers relative to one another (**Extended Data Fig. 8a**).

Our data also do not establish whether a DSB can be religated. Here and elsewhere[24] we propose that the conformation of both yeast and mouse SPO11 is different in the post-DSB vs. pre-DSB state, at least in part through relative motion of the WH and Toprim domains (**Extended Data Fig. 5g**). Moreover, the post-DSB conformation appears incompatible with dimerization because of steric clashes between the two end-bound complexes[24]. If correct, then a post-DSB conformation would also be incompatible with religation because religation would

require reestablishment of a dimer interface similar to the pre-DSB state. In contrast, we suggest that nicking would not allow the covalently bound monomer to relax into the post-DSB configuration without dissociation of the noncovalently bound monomer. This interpretation would allow for nicks to be resealable even if DSBs are not.

4. Sequence preferences in vitro and in vivo

A corollary of our proposal that SPO11 is highly dependent on clustering in vivo is that this clustering should also diminish the dependency of DSB formation on an optimal DNA sequence. This may help to explain why sequence signatures of preferred cleavage sites in vivo are weaker than in vitro (**Fig. 4d**). Moreover, the DSB frequency at a given genomic location is determined by many factors operating over different size scales (e.g., targeting by the PRDM9 histone methyltransferase; nucleosome occupancy; higher order chromosome structure; replication timing, etc.)[37,41,76-79]. These factors shape DSB locations independent of the intrinsic affinity of SPO11 for the DNA at the cut site, so they also are expected to weaken the contribution of SPO11 itself.

5. Relationship between nicking and double-strand cleavage

The relationship between nicking and DSB formation is not currently clear. Nicked products were observed under all of the conditions tested, they tended to appear earlier than DSBs on both plasmid and oligonucleotide substrates, and nicks appeared to be favored under at least some conditions where double-strand cleavage was suboptimal (e.g., with Mg^{2+} or Ca^{2+} instead of Mn^{2+}). One possibility is that cleavage of the two strands is mostly or completely independent, with the rate and efficiency of each strand's cleavage being influenced by the DNA sequence and other factors (e.g., topological stress). An alternative is that first- and second-strand cleavages occur with markedly different rates, e.g., with second-strand cleavage occurring more quickly to yield DSBs in a concerted manner. In either scenario, accumulation of nicked products might reflect SPO11 binding to DNA sequences that are susceptible to first-strand cleavage but suboptimal for second-strand cleavage. Alternatively, there may be a population of defective SPO11–TOP6BL complexes in the protein preparations, which can support nicking by a "normal" dimer partner but cannot support double-strand cleavage. We cannot exclude this alternative, but the observation that the large majority of dimer-bound oligonucleotide substrates acquire a DSB rather than a nick (**Fig. 4g**) argues against this being a major contributor to nick formation in our studies.

6. Efficiency of DNA cleavage in vitro

The plasmid reactions were inefficient on a per-protein basis. For example, even though most of the substrate was cut by 30 min with 100 nM protein in **Fig. 2a**, this represented less than ~300 fmol of strand breaks and thus only ~4% of the SPO11 molecules. With the oligonucleotide substrate and wild-type protein, 72% of the DNA was cut by 60 min (**Fig. 4g**). The fraction of DNA expected to be dimer-bound under the simplistic assumption of two independent monomer binding sites, each with a K_d of 1.8 nM, is the same (72%). This suggests that nearly every SPO11 dimer is capable of making a strand break on the oligonucleotide substrate. We infer from this high apparent efficiency that most or all of the protein preparation is active if it is able to assemble a dimer on a cleavage-susceptible sequence. We also note that there was little or no indication from the EMSAs that an appreciable population of higher order complexes contained a SPO11 dimer plus two DNA duplexes. Thus, our findings do not provide support for binding of a second duplex being required for cleavage[25,33,38], although we do not exclude that such binding can occur and might affect cleavage in other contexts.

7. SPO11 association with REC114–MEI4 complexes

In yeast, Rec114 and Mei4 form a 2:1 heterotrimer, and structural modeling suggests the same is true for the mouse proteins[80,81]. Given the interaction between mouse REC114 and TOP6BL[59], it is thus possible that the presence of two TOP6BL binding sites within a REC114-MEI4 heterotrimer might be expected to promote SPO11 dimer formation. However, current models suggest that the active form of REC114-MEI4 in vivo consists of higher order assemblies with many copies of the trimer, so there is no reason a priori to favor the idea that the two SPO11–TOP6BL complexes that make up a catalytically active dimer must both be bound to the same REC114-MEI4 heterotrimer. Also, it is not yet clear that the SPO11-REC114 interaction is required for DSB catalysis. Moreover, the surface on REC114 that interacts with TOP6BL also interacts with ANKRD31 and IHO1[59,82,83], and there are long unstructured segments connecting the REC114-TOP6BL interaction domains both to SPO11 and to MEI4 and the other copy of REC114 in a trimer. These features also call into question whether a strict stoichiometric relationship exists between REC114-MEI4 trimers and SPO11–TOP6BL dimers.

Supplementary References

65 Yeh, H. Y., Lin, S. W., Wu, Y. C., Chan, N. L. & Chi, P. Functional characterization of the meiosis-specific DNA double-strand break inducing factor SPO-11 from *C. elegans*. *Scientific reports* **7**, 2370 (2017). https://doi.org/10.1038/s41598-017-02641-z

66 An, X. J., Deng, Z. Y. & Wang, T. OsSpo11-4, a rice homologue of the archaeal TopVIA protein, mediates double-strand DNA cleavage and interacts with OsTopVIB. *PLoS One* **6**, e20327 (2011). https://doi.org/10.1371/journal.pone.0020327

67 Shingu, Y., Mikawa, T., Onuma, M., Hirayama, T. & Shibata, T. A DNA-binding surface of SPO11-1, an Arabidopsis SPO11 orthologue required for normal meiosis. *FEBS J* **277**, 2360-2374 (2010). https://doi.org/10.1111/j.1742-4658.2010.07651.x

68 Wu, H., Gao, J., Sharif, W. D., Davidson, M. K. & Wahls, W. P. Purification, folding, and characterization of Rec12 (Spo11) meiotic recombinase of fission yeast. *Protein Expr Purif* **38**, 136-144 (2004). https://doi.org/10.1016/j.pep.2004.07.012

69 Chen, H. W. *et al.* Biochemical characterization of the meiosis-essential yet evolutionarily divergent topoisomerase VIB-like protein MTOPVIB from Arabidopsis thaliana. *Nucleic Acids Res* **52**, 4541-4555 (2024). https://doi.org/10.1093/nar/gkae181

70 Arter, M. & Keeney, S. Divergence and conservation of the meiotic recombination machinery. *Nat Rev Genet* **25**, 309-325 (2024). https://doi.org/10.1038/s41576-023-00669-8

71 Jolivet, S., Vezon, D., Froger, N. & Mercier, R. Non conservation of the meiotic function of the Ski8/Rec103 homolog in *Arabidopsis*. *Genes to Cells* **11**, 615-622 (2006). https://doi.org/10.1111/j.1365-2443.2006.00972.x

72 Schmidt, B. H., Burgin, A. B., Deweese, J. E., Osheroff, N. & Berger, J. M. A novel and unified two-metal mechanism for DNA cleavage by type II and IA topoisomerases. *Nature* **465**, 641-644 (2010). https://doi.org/10.1038/nature08974

73 Deweese, J. E., Burgin, A. B. & Osheroff, N. Human topoisomerase IIalpha uses a two-metal-ion mechanism for DNA cleavage. *Nucleic Acids Res* **36**, 4883-4893 (2008). https://doi.org/10.1093/nar/gkn466

74 Pitts, S. L. *et al.* Use of divalent metal ions in the DNA cleavage reaction of topoisomerase IV. *Nucleic Acids Res* **39**, 4808-4817 (2011). https://doi.org/10.1093/nar/gkr018

75 Diaz, R. L., Alcid, A. D., Berger, J. M. & Keeney, S. Identification of residues in yeast Spo11p critical for meiotic DNA double-strand break formation. *Mol Cell Biol* **22**, 1106-1115 (2002). https://doi.org/10.1128/MCB.22.4.1106-1115.2002

76 Tischfield, S. E. & Keeney, S. Scale matters: the spatial correlation of yeast meiotic DNA breaks with histone H3 trimethylation is driven largely by independent colocalization at promoters. *Cell cycle* **11**, 1496-1503 (2012). https://doi.org/10.4161/cc.19733

77 Murakami, H. *et al.* Multilayered mechanisms ensure that short chromosomes recombine in meiosis. *Nature* **582**, 124-128 (2020). https://doi.org/10.1038/s41586-020-2248-2

78 Brick, K., Smagulova, F., Khil, P., Camerini-Otero, R. D. & Petukhova, G. V. Genetic recombination is directed away from functional genomic elements in mice. *Nature* **485**, 642-645 (2012). https://doi.org/10.1038/nature11089

79 Pratto, F. *et al.* Meiotic recombination mirrors patterns of germline replication in mice and humans. *Cell* **184**, 4251-4267 e4220 (2021). https://doi.org/10.1016/j.cell.2021.06.025

80 Liu, K. *et al.* Structure and DNA-bridging activity of the essential Rec114-Mei4 trimer interface. *Genes Dev* **37**, 518-534 (2023). https://doi.org/10.1101/gad.350461.123

81 Daccache, D. *et al.* Evolutionary conservation of the structure and function of meiotic Rec114-Mei4 and Mer2 complexes. *Genes Dev* **37**, 535-553 (2023). https://doi.org/10.1101/gad.350462.123

82 Laroussi, H. *et al.* Characterization of the REC114-MEI4-IHO1 complex regulating meiotic DNA double-strand break formation. *EMBO J* **42**, e113866 (2023). https://doi.org/10.15252/embj.2023113866

83 Boekhout, M. *et al.* REC114 partner ANKRD31 controls number, timing, and location of meiotic DNA breaks. *Mol Cell* **74**, 1053-1068 e1058 (2019). https://doi.org/10.1016/j.molcel.2019.03.023

Supplementary Table 1. DNA sequences for binding and cleavage substrates and AlphaFold 3 modeling

**a. DNA substrates for EMSAs**	
25 bp hairpin	5′-TAGCAATGTAATCGTCTATGACGTTAACGTCATAGACGATTACATTGC-3′
32 bp, top	5′-TAGCAATGTAATCGTCTATGACGTGTCATAGCGC-3′
32 bp, bottom	5′-TAGCGCTATGACACGTCATAGACGATTACATTGC-3′
31 bp, top	5′-TAGCAATGTAATCGTCTATGACGTGCATAGCGC-3′
31 bp, bottom	5′-TAGCGCTATGCACGTCATAGACGATTACATTGC-3′
30 bp, top	5′-TAGCAATGTAATCGTCTATGACGTGATAGCGC-3′
30 bp, bottom	5′-TAGCGCTATCACGTCATAGACGATTACATTGC-3′
29 bp, top	5′-TAGCAATGTAATCGTCTATGACGTGTAGCGC-3′
29 bp, bottom	5′-TAGCGCTACACGTCATAGACGATTACATTGC-3′
28 bp, top	5′-TAGCAATGTAATCGTCTATGACGTTAGCGC-3′
28 bp, bottom	5′-TAGCGCTAACGTCATAGACGATTACATTGC-3′
27 bp, top	5′-TAGCAATGTAATCGTCTATGACGTTAGCG-3′
27 bp, bottom	5′-TACGCTAACGTCATAGACGATTACATTGC-3′
26 bp, top	5′-TAGCAATGTAATCGTCTATGACGTTAGC-3′
26 bp, bottom	5′-TAGCTAACGTCATAGACGATTACATTGC-3′
25 bp, top	5′-TAGCAATGTAATCGTCTATGACGTTAG-3′
25 bp, bottom	5′-TACTAACGTCATAGACGATTACATTGC-3′
24 bp, top	5′-TAGCAATGTAATCGTCTATGACGTTA-3′
24 bp, bottom	5′-TATAACGTCATAGACGATTACATTGC-3′
23 bp, top	5′-TAGCAATGTAATCGTCTATGACGTT-3′
23 bp, bottom	5′-TAAACGTCATAGACGATTACATTGC-3′
22 bp, top	5′-TAGCAATGTAATCGTCTATGACGT-3′
22 bp, bottom	5′-TAACGTCATAGACGATTACATTGC-3′
21 bp, top	5′-TAGCAATGTAATCGTCTATGACG-3′
21 bp, bottom	5′-TACGTCATAGACGATTACATTGC-3′

**b. DNA sequences for AlphaFold3 (top strands only)**	
36 bp, seq 1 *	5′-GTTCGTTGTTACGAAGCATACCCAAACACTTCCCTA-3′
36 bp, seq 2	5′-GCTAGGCAAAAATGGGCATGCCTTTTCTCCATTAAT-3′
36 bp, seq 3	5′-TAGAATCTTGTGTTGGTATACCTCTATATACTAATA-3′
40 bp	5′-GTGTTCGTTGTTACGAAGCATACCCAAACACTTCCCTACC-3′
44 bp	5′-CGGTGTTCGTTGTTACGAAGCATACCCAAACACTTCCCTACCAC-3′

c. DNA sequence for oligonucleotide cleavage assay**

↓

5′-CGGTTCAAAAGAACCGGAGCTGAATGAAGCC.ATACCAAACGACGAGCGTGACACAAAAGTGTC
ACGCTCGTCGTTTGGT.ATGGCTTCATTCAGCTC-3′

↑

* The top model generated with this DNA was used in the figures (Supplementary File 1).

** A4 loops underlined; central 6 bp around preferred cleavage site from pUC19 in red; arrows, predicted cleavage positions.

Supplementary Table 2. TDP2-seq mapping statistics

Library	No. of reads	No. mapped*	No. uniquely mapped*	No. mapped (mm10)**	No. uniquely mapped (mm10)**
pUC19 DNA, SPO11-WT, rep 1	26,446,950	3762	3584	2,892,802	1,212,280
pUC19 DNA, SPO11-WT, rep 2	29,036,360	4710	4544	2,580,520	1,041,174
pUC19 DNA, SPO11-Y138F, rep 1	26,460,313	1342	1284	3,226,714	1,375,254
pUC19 DNA, SPO11-Y138F, rep 2	24,815,713	1246	1214	2,462,532	947,926
E. coli DNA, SPO11-WT, rep 1	15,211,454	409,778	393,176	7,642,192	1,828,402
E. coli DNA, SPO11-WT, rep 2	25,379,538	508,514	483,160	12,734,598	3,129,618
E. coli DNA, SPO11-Y138F, rep 1	24,380,052	472,132	449,016	3,954,258	2,674,612
E. coli DNA, SPO11-Y138F, rep 2	19,787,344	398,098	377,752	10,068,448	2,467,122
S. cerevisiae DNA, SPO11-WT, rep 1	12,338,811	290,444	230,234	4,744,010	1,473,164
S. cerevisiae DNA, SPO11-WT, rep 2	12,525,951	473,424	372,610	4,470,974	1,452,914
S. cerevisiae, SPO11-Y138F, rep 1	9,191,207	231,826	171,504	4,302,632	1,322,228
S. cerevisiae, SPO11-Y138F, rep 2	7,974,490	241,740	176,736	4,221,372	1,295,052
Mre11-cKO mouse testis, rep 1	93,285,814			40,041,912	10,032,498
Mre11-cKO mouse testis, rep 2	60,567,142			24,414,746	5,874,994

* Reads mapped to pUC19 sequence (pUC19 samples), ASM584v2 genome assembly (*E. coli* samples) or sacCer3 genome assembly (*S. cerevisiae* samples), respectively.

** Reads mapped to mouse mm10 genome assembly. For the in vitro cleavage reactions, these reads arose from mouse cells spiked in as carrier during agarose plug preparation (Methods).

Supplementary Figure 1: Gel source data

Figure 1b

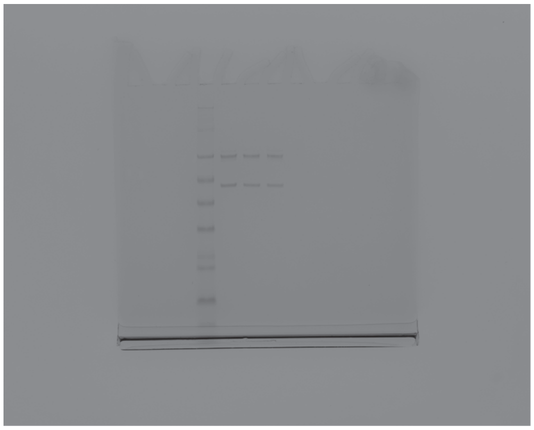

Figure 1d

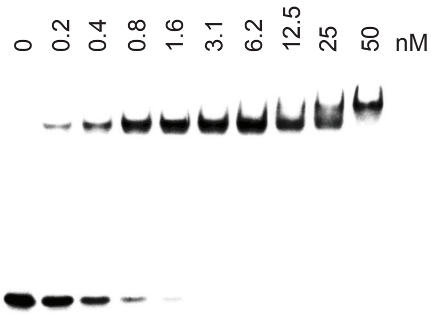

Figure 1e-28 bp

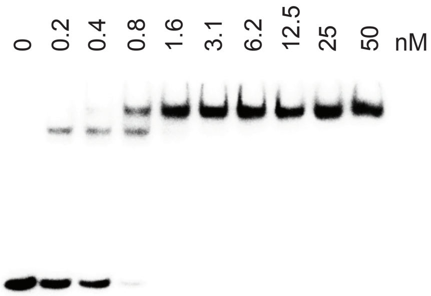

Figure 1e-24 bp

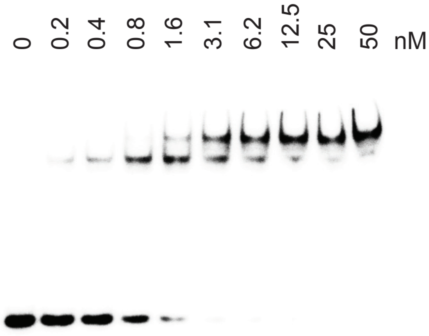

Figure 1e-22 bp

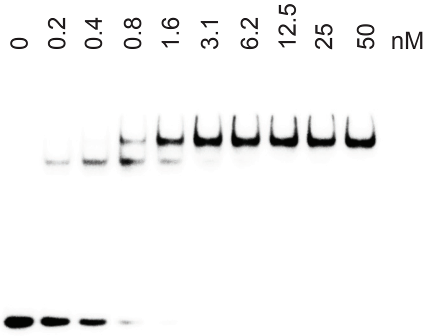

Figure 1f-one end

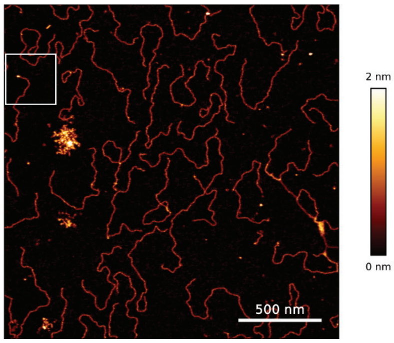

Figure 1f-duplex

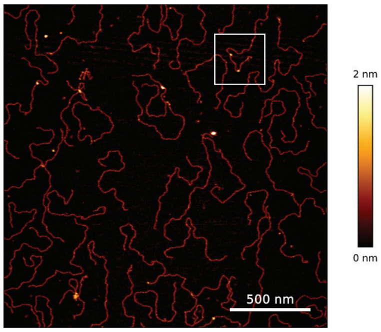

Figure 1f-three-way

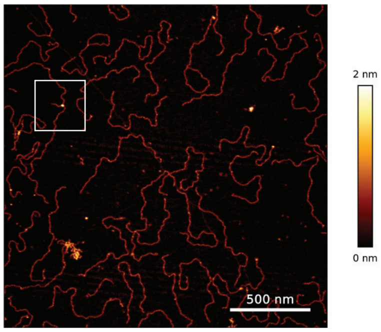

Figure 1f-four-way

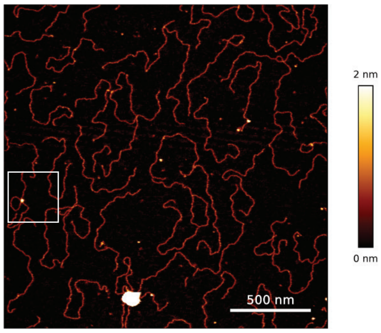

Figure 1g-60 degrees

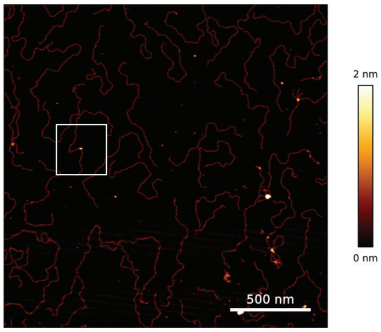

Figure 1g-120 degrees

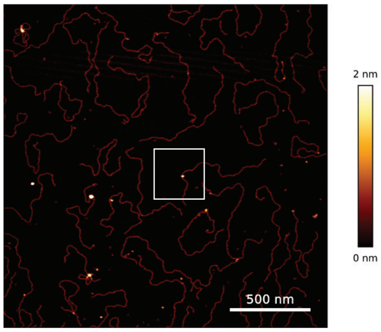

Supplementary Figure 1: Gel source data (continued)

Figure 2a

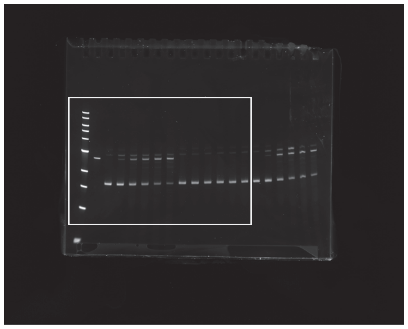

Figure 2c-fair

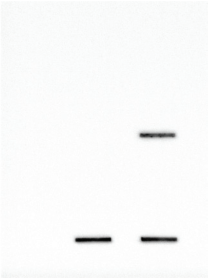

Figure 2c-saturated

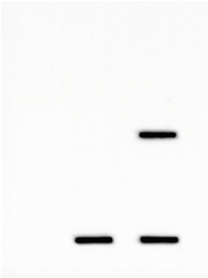

Figure 2d

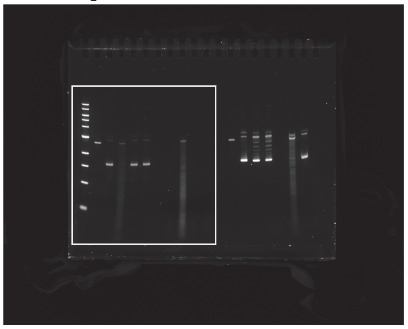

Figure 2f

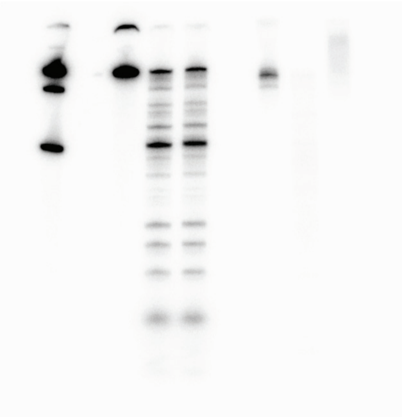

Figure 2h-simultaneous, P1-P2

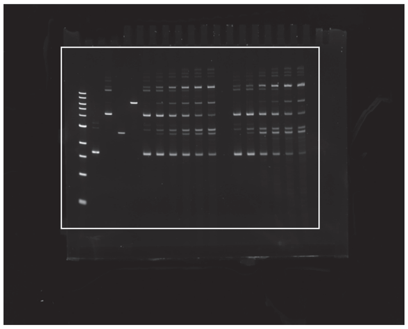

Figure 2h-P2-P1

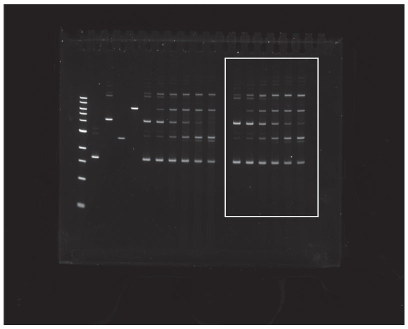

Figure 3g-YF

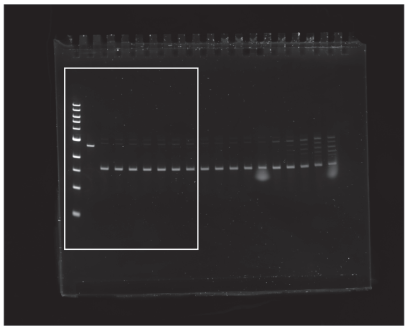

Figure 3g-EA, YF+EA

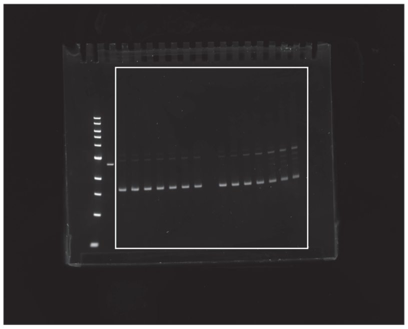

Figure 3h-No EtBr

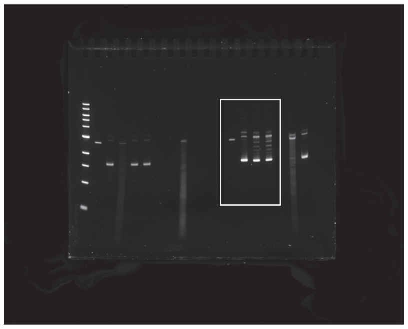

Figure 3h-+ EtBr

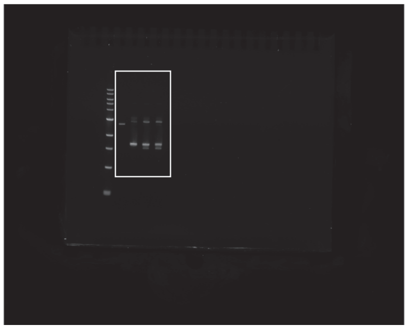

Figure 4g

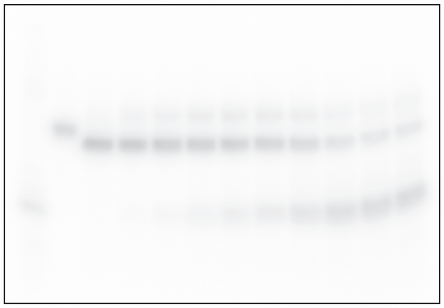

Figure 4h

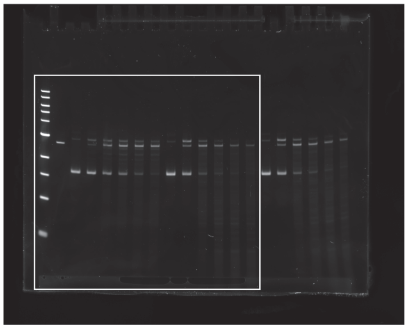

Supplementary Figure 1: Gel source data (continued)

ED Figure 1a

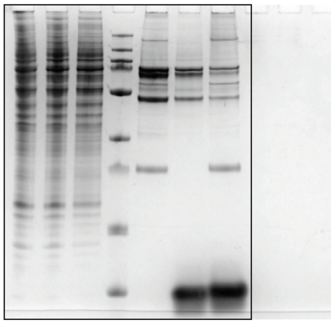

ED Figure 1b

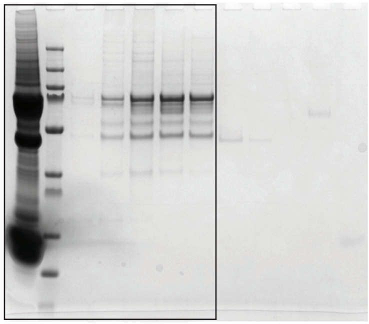

ED Figure 1b

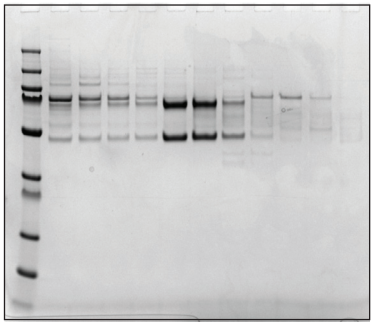

ED Figure 1c

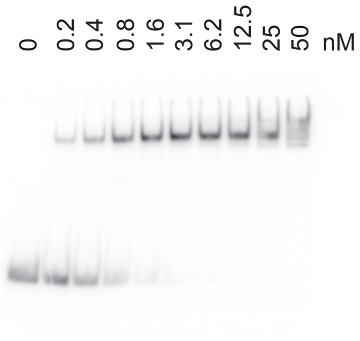

ED Figure 1d

22/24/28 bp figures are already in source data-Fig. 1

ED Figure 1d-21 bp

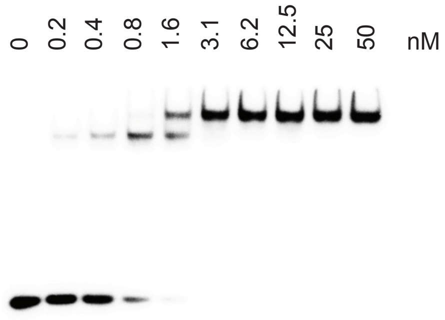

ED Figure 1d-23 bp

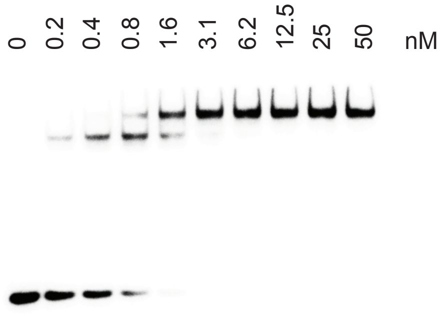

ED Figure 1d-25 bp

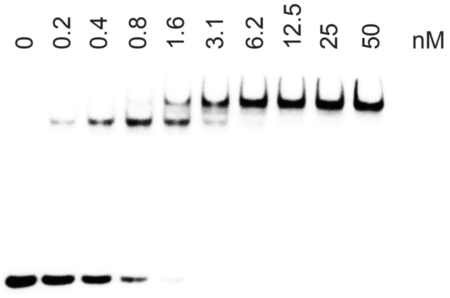

ED Figure 1d-26 bp

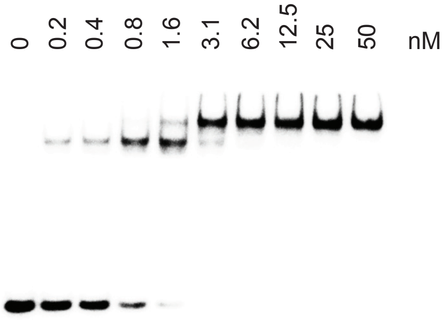

ED Figure 1d-27 bp

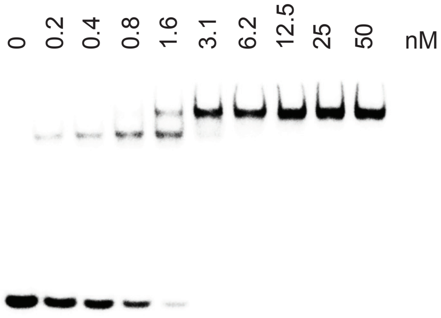

ED Figure 1d-29 bp

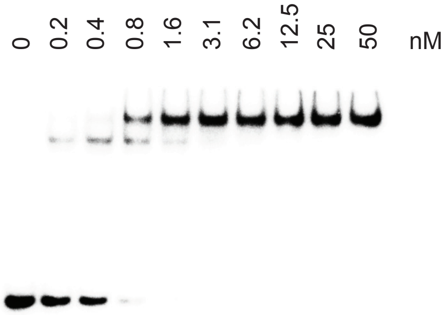

ED Figure 1d-30 bp

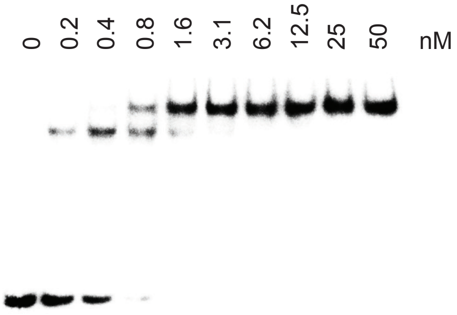

ED Figure 1d-31 bp

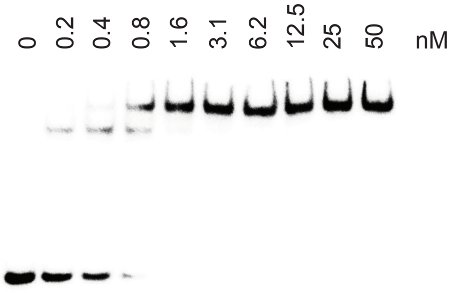

ED Figure 1d-32 bp

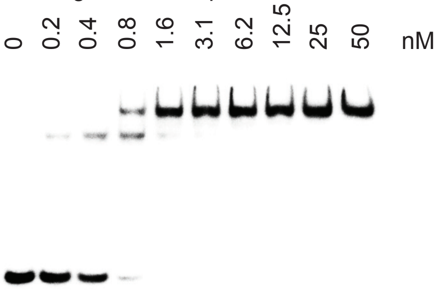

Supplementary Figure 1: Gel source data (continued)

ED Figure 2a

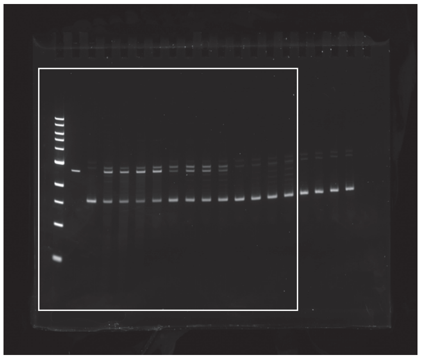

ED Figure 2b

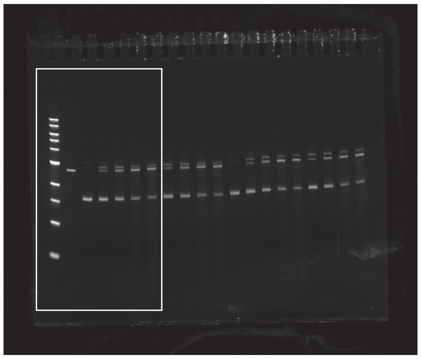

ED Figure 2c

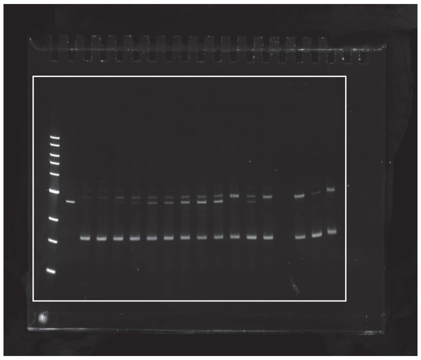

ED Figure 2d

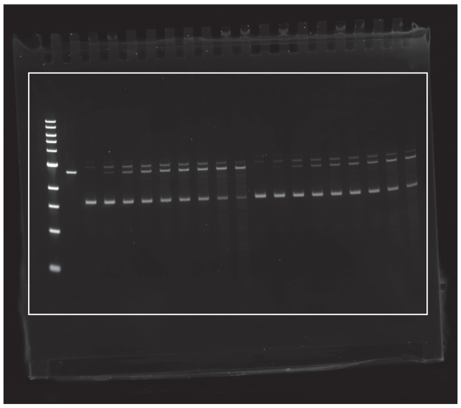

ED Figure 2e

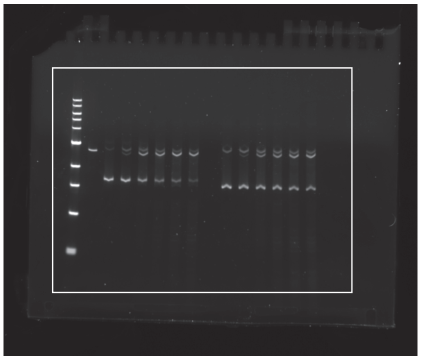

ED Figure 2f-native

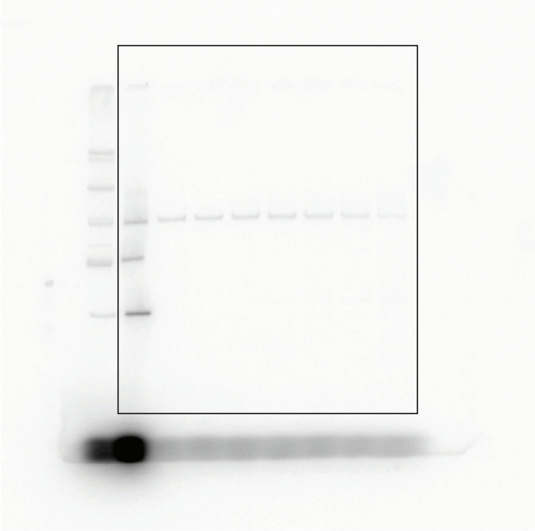

ED Figure 2f-denature

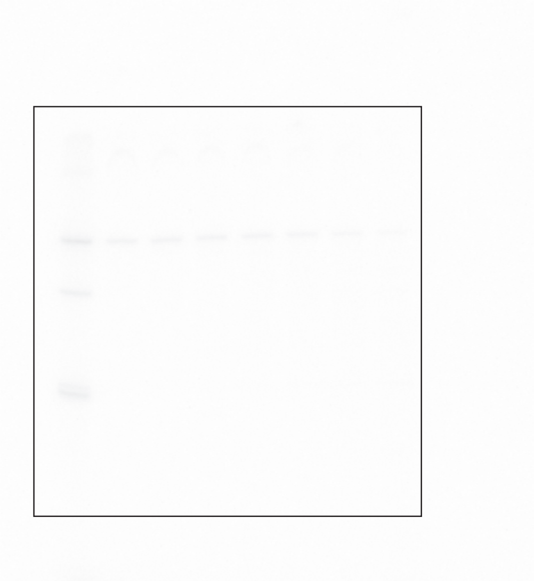

Supplementary Figure 1: Gel source data (continued)

ED Figure 5h

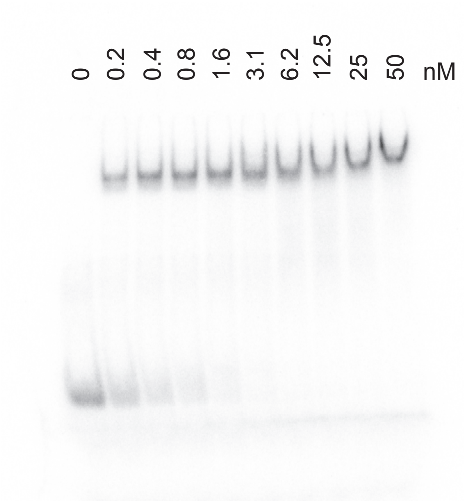

ED Figure 5i-No EtBr

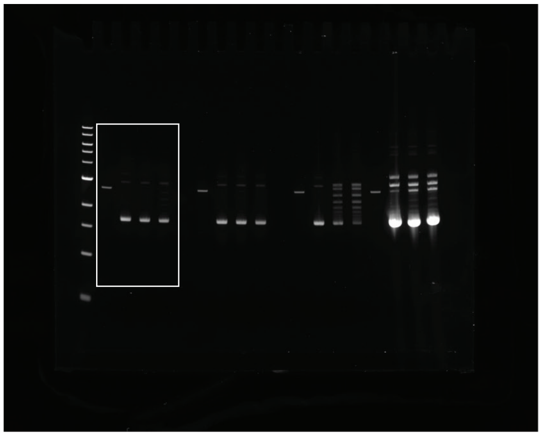

ED Figure 5i-with EtBr

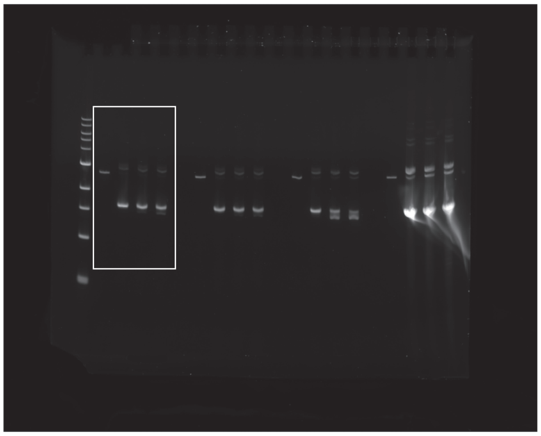

ED Figure 7a

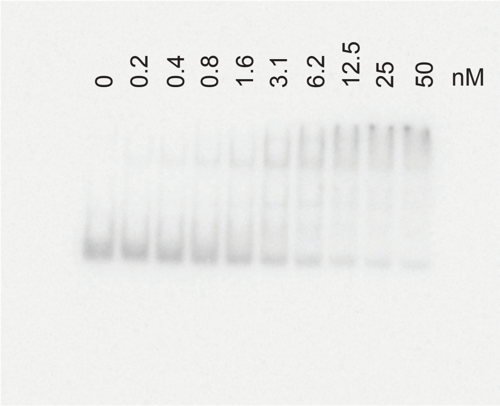

ED Figure 7b

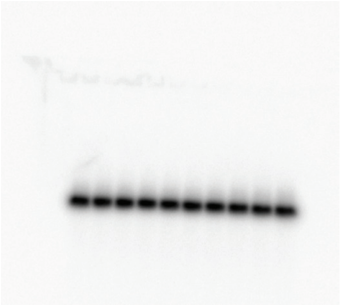

ED Figure 7c

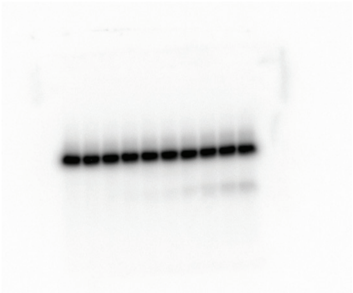

ED Figure 7f-FKBP-SPO11

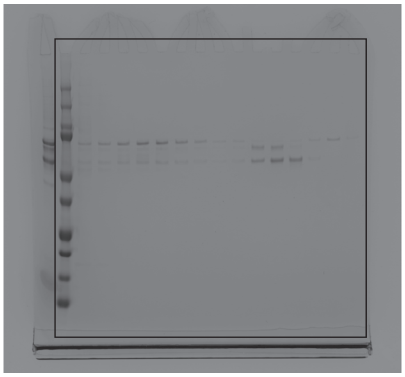

ED Figure 7d

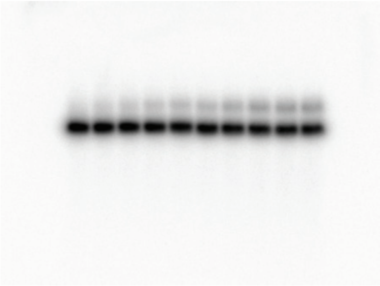

ED Figure 7e

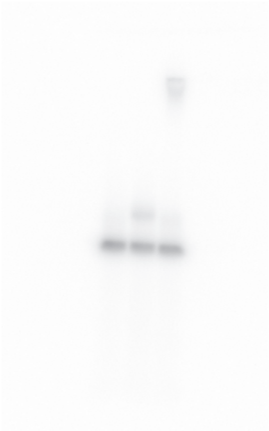

ED Figure 7f-FRB-SPO11

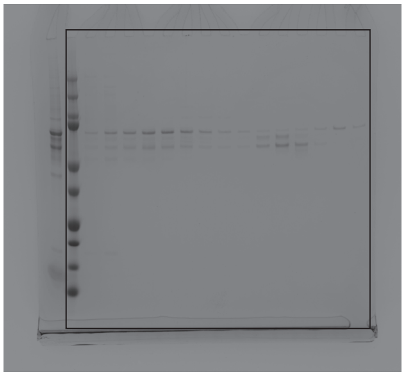

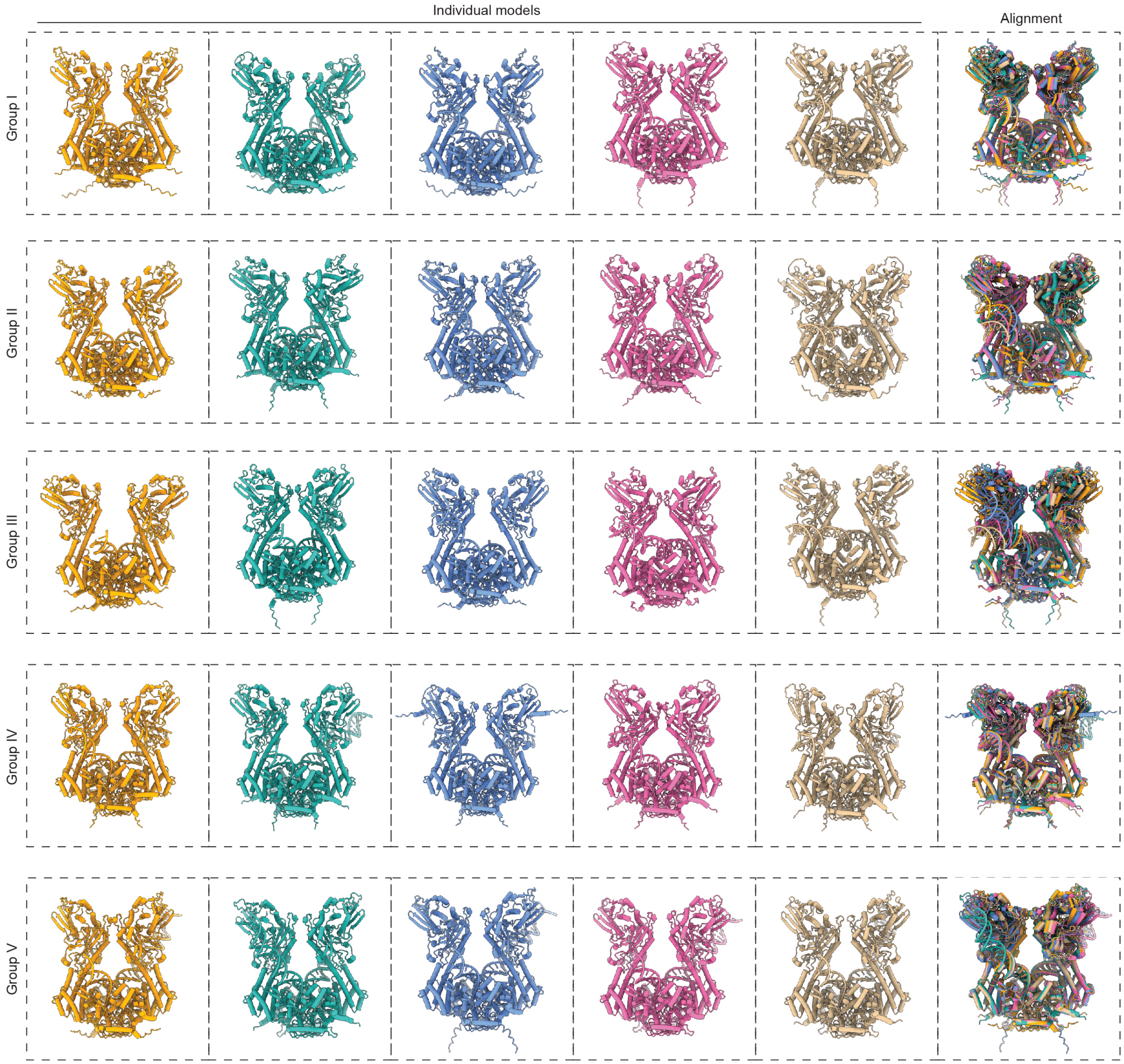

Supplementary Fig. 2 | Gallery of AlphaFold3 models. The top five AlphaFold3 models are shown for each of the five DNA sequences shown in Supplementary Table 1b.
