# Supplementary material for: Reconstitution of SPO11-dependent double-strand break formation
Source: Nature. 2025 Feb 19;639(8055):784–91. doi: 10.1038/s41586-025-08601-2 (PMC11922745; doi:10.1038/s41586-025-08601-2)
Supplement: Supplementary file 2 — Reporting Summary [file 41586_2025_8601_MOESM2_ESM.pdf]

nature portfolio

Corresponding author(s): Scott Keeney

Last updated by author(s): Nov 29, 2024

Reporting Summary

Nature Portfolio wishes to improve the reproducibility of the work that we publish. This form provides structure for consistency and transparency in reporting. For further information on Nature Portfolio policies, see our Editorial Policies and the Editorial Policy Checklist.

Statistics

For all statistical analyses, confirm that the following items are present in the figure legend, table legend, main text, or Methods section.

n/a	Confirmed	
☐	☒	The exact sample size (*n*) for each experimental group/condition, given as a discrete number and unit of measurement
☐	☒	A statement on whether measurements were taken from distinct samples or whether the same sample was measured repeatedly
☒	☐	The statistical test(s) used AND whether they are one- or two-sided<br>*Only common tests should be described solely by name; describe more complex techniques in the Methods section.*
☒	☐	A description of all covariates tested
☒	☐	A description of any assumptions or corrections, such as tests of normality and adjustment for multiple comparisons
☐	☒	A full description of the statistical parameters including central tendency (e.g. means) or other basic estimates (e.g. regression coefficient) AND variation (e.g. standard deviation) or associated estimates of uncertainty (e.g. confidence intervals)
☒	☐	For null hypothesis testing, the test statistic (e.g. *F*, *t*, *r*) with confidence intervals, effect sizes, degrees of freedom and *P* value noted<br>*Give P values as exact values whenever suitable.*
☒	☐	For Bayesian analysis, information on the choice of priors and Markov chain Monte Carlo settings
☒	☐	For hierarchical and complex designs, identification of the appropriate level for tests and full reporting of outcomes
☒	☐	Estimates of effect sizes (e.g. Cohen's *d*, Pearson's *r*), indicating how they were calculated

Our web collection on statistics for biologists contains articles on many of the points above.

Software and code

Policy information about availability of computer code

Data collection	Mass Photometry: Refeyn AcquireMP (version 2024.1.1.0)<br>Atomic force microscopy: JPK Scanning Probe Microscope Control Program (version 8.0.59.1)<br>Phosphor Imaging scan: Amersham typhoon control software (version 4.0.0.4)<br>SYBR Gold Scan: BioRad Image Lab Touch Software (version 3.0.1.14)<br>Illumina sequencing: Real Time Analysis (version 3.1 or version 4.1)<br>Size exclusion chromatography: UNICORN 7.6 (version Build 7.6.0.1306)<br>A260/280 for protein quantification: NanoDrop 8000 (version 2.3.3)<br>AlphaFold3 (server: https://alphafoldserver.com)<br>UNICORN 7.6 (version Build 7.6.0.1306)<br>NanoDrop 8000 (version 2.3.3)<br>Amersham typhoon control software (version 4.0.0.4)
Data analysis	GelBandFitter (version 1.7)<br>GraphPad Prism 10 (version 10.3.0 (461) for Mac OS X)<br>Image Lab (version 6.1.0 build 7)<br>ImageJ (version 1.54g)<br>DRAGEN suite (version 4.2.7)<br>bowtie2 (version 2.5.3)<br>ggseqlogo (version 0.2)<br>European Molecular Biology Open Software Suite [EMBOSS] (version 6.6.0)<br>R (versions 4.2.3 and 4.3.2)

Chimera (version 1.18)
ChimeraX (version 1.8)
Refeyn DiscoverMP (version 2024.1.0.0)
JPK Data Processing Software (version 8.0.59.1)

For manuscripts utilizing custom algorithms or software that are central to the research but not yet described in published literature, software must be made available to editors and reviewers. We strongly encourage code deposition in a community repository (e.g. GitHub). See the Nature Portfolio guidelines for submitting code & software for further information.

Data

Policy information about availability of data

All manuscripts must include a data availability statement. This statement should provide the following information, where applicable:

- Accession codes, unique identifiers, or web links for publicly available datasets
- A description of any restrictions on data availability
- For clinical datasets or third party data, please ensure that the statement adheres to our policy

Raw and processed TDP2-seq data are available at GEO under accession number GSE275291 (https://www.ncbi.nlm.nih.gov/geo/query/acc.cgi?acc=GSE275291). The AlphaFold3 model used to generate most of the figures is provided in .pdb format as Supplemental Data. The mouse genome assembly mm10 (a.k.a. GRCm38) is available at https://www.ncbi.nlm.nih.gov/datasets/genome/GCF_000001635.20/

Research involving human participants, their data, or biological material

Policy information about studies with human participants or human data. See also policy information about sex, gender (identity/presentation), and sexual orientation and race, ethnicity and racism.

Reporting on sex and gender	n/a, no human subjects data
Reporting on race, ethnicity, or other socially relevant groupings	n/a, no human subjects data
Population characteristics	n/a, no human subjects data
Recruitment	n/a, no human subjects data
Ethics oversight	n/a, no human subjects data

Note that full information on the approval of the study protocol must also be provided in the manuscript.

Field-specific reporting

Please select the one below that is the best fit for your research. If you are not sure, read the appropriate sections before making your selection.

☒ Life sciences ☐ Behavioural & social sciences ☐ Ecological, evolutionary & environmental sciences

For a reference copy of the document with all sections, see nature.com/documents/nr-reporting-summary-flat.pdf

Life sciences study design

All studies must disclose on these points even when the disclosure is negative.

Sample size	All DNA cleavage experiments were performed in triplicate unless stated otherwise. This sample size was chosen because preliminary experiments determined it to be sufficient to distinguish effect sizes deemed to be meaningful for purposes of the studies presented. Sequencing experiments were conducted in two replicates (biological replicates for in vivo data). Prior published work (https://pubmed.ncbi.nlm.nih.gov/39149289/) has established that two replicates are sufficient to determine reproducibility because of the high correlation between replicates for these methods.
Data exclusions	For sequencing data: sequence reads mapping at chromosome ends were excluded because they reflect natural end structure and not SPO11-dependent cleavage; sequence reads mapping to repetitive DNA were excluded because they cannot be assigned with confidence to specific locations, and our previous published work has established that excluding them does not affect the conclusions drawn. No other data were excluded.
Replication	All in vitro DNA cleavage experiments were repeated at least twice with similar results. The protein purifications were repeated at least twice, and for wild type proteins more than five times each, with similar results. DNA sequencing experiments were performed in two replicates each and assessed for reproducibility by direct quantitative comparison; all sequencing experiments were successfully replicated.
Randomization	Experiments involved comparison of mutants to matched wild-type controls, or comparison of the same protein's behaviors under different reaction conditions, so randomization is neither necessary nor appropriate.

Blinding	No blinding was used. All biochemical experiments involved comparison of wild type with mutant proteins, or comparison of different in vitro conditions for the same protein. In vivo experiments involved independent replicates performed on mice of the same genotype. It is not standard practice in the field to use blinding for these assays. Moreover, blinding is not necessary with this experimental design because meaningful effect sizes are larger than any likely effects of operator bias.

Reporting for specific materials, systems and methods

We require information from authors about some types of materials, experimental systems and methods used in many studies. Here, indicate whether each material, system or method listed is relevant to your study. If you are not sure if a list item applies to your research, read the appropriate section before selecting a response.

Materials & experimental systems

n/a	Involved in the study
☐	☒ Antibodies
☐	☒ Eukaryotic cell lines
☒	☐ Palaeontology and archaeology
☐	☒ Animals and other organisms
☒	☐ Clinical data
☒	☐ Dual use research of concern
☒	☐ Plants

Methods

n/a	Involved in the study
☒	☐ ChIP-seq
☒	☐ Flow cytometry
☒	☐ MRI-based neuroimaging

Antibodies

Antibodies used	anti-Flag M2 affinity gel (Sigma A2220)<br>anti-Flag-HRP monoclonal antibody (mouse, 1:1000, Sigma A8592)<br>anti-Flag magnetic agarose (Pierce A36797)
Validation	Antibody specificity was confirmed by data shown in the paper, namely, successful affinity purification of recombinant Flag-tagged SPO11 complexes in purifications and immunoprecipitations, and absence of detectable signal in negative controls in immunoblotting experiments.

Eukaryotic cell lines

Policy information about cell lines and Sex and Gender in Research

Cell line source(s)	FreeStyle™ 293-F cells, Invitrogen
Authentication	The cell lines were not authenticated
Mycoplasma contamination	Cell lines were not tested for mycoplasma.
Commonly misidentified lines<br>(See ICLAC register)	None

Animals and other research organisms

Policy information about studies involving animals; ARRIVE guidelines recommended for reporting animal research, and Sex and Gender in Research

Laboratory animals	Mus musculus, young adult (<4 mos old) Mre11 conditional knockout mice (Mre11-flox/del Ngn3-Cre on a congenic C57BL/6J background) were used.
Wild animals	The study did not involve wild animals
Reporting on sex	Experiments analyzed spermatogenesis, so only male mice were used.
Field-collected samples	The study did not involve samples collected from the field.
Ethics oversight	Mouse experiments were performed in accordance with US Office of Laboratory Animal Welfare regulations and were approved by the Memorial Sloan Kettering Cancer Center Institutional Animal Care and Use Committee.

Note that full information on the approval of the study protocol must also be provided in the manuscript.

Plants

Seed stocks	*Report on the source of all seed stocks or other plant material used. If applicable, state the seed stock centre and catalogue number. If plant specimens were collected from the field, describe the collection location, date and sampling procedures.*
Novel plant genotypes	*Describe the methods by which all novel plant genotypes were produced. This includes those generated by transgenic approaches, gene editing, chemical/radiation-based mutagenesis and hybridization. For transgenic lines, describe the transformation method, the number of independent lines analyzed and the generation upon which experiments were performed. For gene-edited lines, describe the editor used, the endogenous sequence targeted for editing, the targeting guide RNA sequence (if applicable) and how the editor was applied.*
Authentication	*Describe any authentication procedures for each seed stock used or novel genotype generated. Describe any experiments used to assess the effect of a mutation and, where applicable, how potential secondary effects (e.g. second site T-DNA insertions, mosiacism, off-target gene editing) were examined.*
