# Supplementary material for: Reconstitution of SPO11-dependent double-strand break formation
Source: Nature. 2025 Feb 19;639(8055):784–91. doi: 10.1038/s41586-025-08601-2 (PMC11922745; doi:10.1038/s41586-025-08601-2)
Supplement: Supplementary file 5 — Protein sequences [file 41586_2025_8601_MOESM5_ESM.pdf]

Amino acid sequences of recombinant proteins

Flag-TEV-SPO11:

MDYKDDDDKENLYFQGAFAPMGPEASFFDALDRHRASLLAMVKRGAGETPAGATRVA
SSSEVLTAIENIIQDIIKSLARNEVPAFTIDNRSSWENIMFDDSVGLRMIPQCTTRKIRSDS
PKSVKKFALILKVLSMIYKLIQSDTYATKRDIYYTDSQLFGNQAAVDSAIDDISCMLKVPR
RSLHVLSTSKGLIAGNLRYMEEDGTRVQCTCSATATAVPTNIQGMQHLITDAKFLLIVEK
DATFQRLLDDNFCSRMSPCIMVTGKGVPDLNTRLLVKKLWDTFHIPVFTLVDADPYGIEI
MCIYKYGSMSMSFEAHNLTIPTIRWLGLLPSDIQRLNIPKDSLIPLTKHDQMKLDSILKRP
YITYQPLWKKELEMMADSKMKAEIQALTLLSSDYLSRVYLPNKLRFGGWI*

Flag-FKBP-linker-SPO11 fusion:

MDYKDDDDKGVQVETISPGDGRTFPKRGQTCVVHYTGMLEDGKKFDSSRDRNKPFK
FMLGKQEVIRGWEEGVAQMSVGQRAKLTISPDYAYGATGHPGIIPPHATLVFDVELLKL
EGSGSGGGSGGSGGSENLYFQGAFAPMGPEASFFDALDRHRASLLAMVKRGAGETP
AGATRVASSSEVLTAIENIIQDIIKSLARNEVPAFTIDNRSSWENIMFDDSVGLRMIPQCTT
RKIRSDSPKSVKKFALILKVLSMIYKLIQSDTYATKRDIYYTDSQLFGNQAAVDSAIDDISC
MLKVPRRSLHVLSTSKGLIAGNLRYMEEDGTRVQCTCSATATAVPTNIQGMQHLITDAK
FLLIVEKDATFQRLLDDNFCSRMSPCIMVTGKGVPDLNTRLLVKKLWDTFHIPVFTLVDA
DPYGIEIMCIYKYGSMSMSFEAHNLTIPTIRWLGLLPSDIQRLNIPKDSLIPLTKHDQMKL
DSILKRPYITYQPLWKKELEMMADSKMKAEIQALTLLSSDYLSRVYLPNKLRFGGWI*

Flag-FRB-linker-SPO11 fusion:

MDYKDDDDKVAILWHEMWHEGLEEASRLYFGERNVKGMFEVLEPLHAMMERGPQTL
KETSFNQAYGRDLMEAQEWCRKYMKSGNVKDLTQAWDLYYHVFRRISGSGSGGGSG
GSGGSENLYFQGAFAPMGPEASFFDALDRHRASLLAMVKRGAGETPAGATRVASSSE
VLTAIENIIQDIIKSLARNEVPAFTIDNRSSWENIMFDDSVGLRMIPQCTTRKIRSDSPKSV
KKFALILKVLSMIYKLIQSDTYATKRDIYYTDSQLFGNQAAVDSAIDDISCMLKVPRRSLH
VLSTSKGLIAGNLRYMEEDGTRVQCTCSATATAVPTNIQGMQHLITDAKFLLIVEKDATF
QRLLDDNFCSRMSPCIMVTGKGVPDLNTRLLVKKLWDTFHIPVFTLVDADPYGIEIMCIY
KYGSMSMSFEAHNLTIPTIRWLGLLPSDIQRLNIPKDSLIPLTKHDQMKLDSILKRPYITY
QPLWKKELEMMADSKMKAEIQALTLLSSDYLSRVYLPNKLRFGGWI*
